# Obesity and access to kidney transplantation in patients starting dialysis: A prospective cohort study

**DOI:** 10.1371/journal.pone.0176616

**Published:** 2017-05-11

**Authors:** Mathilde Lassalle, Léopold K. Fezeu, Cécile Couchoud, Thierry Hannedouche, Ziad A. Massy, Sébastien Czernichow

**Affiliations:** 1 REIN registry, Agence de la Biomédecine, F-93212 Saint-Denis-la-Plaine cedex, France; 2 Université Paris 13, Equipe de Recherche en Epidémiologie Nutritionnelle (EREN), Centre de Recherche en Epidémiologie et Statistiques, INSERM (U1153), INRA (U1125), CNAM, COMUE Sorbonne Paris Cité, F-93017 Bobigny, France; 3 Department of Nephrology, University of Strasbourg & Hôpitaux Universitaires de Strasbourg, F-67000, Strasbourg, France; 4 Division of Nephrology, Ambroise Paré University Hospital, Assistance Publique—Hôpitaux de Paris, F-92100, Boulogne-Billancourt, France; 5 INSERM U1018, Research Centre in Epidemiology and Population Health (CESP) Team 5, F-94807, Villejuif, France; 6 INSERM UMS 011, F-94807, Villejuif, France; 7 University Paris Descartes, Paris, France; 8 Department of Nutrition, Assistance Publique—Hôpitaux de Paris, Hôpital Européen Georges-Pompidou, Paris, F-75015 France; Hospital Universitario de la Princesa, SPAIN

## Abstract

**Background:**

Obesity has been linked to poor access to medical care. Although scientific evidence suggest that kidney transplantation improves survival and quality of life in obese patients with end-stage renal disease (ESRD), few data exist on the impact of obesity on access to kidney transplantation in this population.

**Objectives:**

We aimed to characterize the relationships between body mass index (BMI) at the start of dialysis, changes in BMI after the start of dialysis, and either access to kidney transplantation or overall mortality in dialysis or transplantation among ESRD patients.

**Methods:**

Between 2002 and 2011, 19524 dialysis patients with ESRD were included in the study via the French nationwide Renal Epidemiology and Information Network. Data on sociodemographic factors, comorbidities and laboratory test results were recorded upon entry into the registry. BMI were obtained at the start of dialysis and then yearly. Cubic spline regression analyses provided a graphic evaluation of the relationships between BMI at the start of dialysis and outcomes. Joint models were used to evaluate the association between the change over time in BMI and outcomes.

**Results:**

During a median follow-up of 20.3 months, 6634 patients underwent kidney transplantation. A BMI >31 kg/m^2^ at the start of the dialysis was associated with a lower likelihood of receiving a kidney transplant, and the likelihood decreased even further with higher BMI values. For patients with BMI ≥30kg/m^2^ at the start of the dialysis, a 1 kg/m^2^ decrease in BMI during follow-up was associated with a 9% to 11% increase in the likelihood of receiving a transplant. There was an L-shaped relationship between BMI at the start of dialysis and overall mortality. We showed that obese patients with ESRD face barriers to the receipt of a kidney transplant without valid reasons.

**Conclusion:**

Greater attention to this issue would improve the fairness of the organ allocation process and might improve outcomes for obese patients with ESRD.

## Introduction

In 2008, 1.5 billion adults worldwide were considered to be overweight, and nearly 500 million of these were clinically obese [1]. Overweight and obesity are established risk factors for a number of chronic conditions, including type II diabetes, cardiovascular disease and various cancers [2, 3]. Moreover, obesity has been associated with less likely to have routine care [4].

As in the general population, obesity has increased in patients with end-stage renal disease (ESRD) [5]. Some (but not all) studies have reported higher survival rates in obese patients with ESRD on maintenance dialysis (the so-called “obesity paradox”) [6]. Among patients with ESRD in general, kidney transplantation improves survival and quality of life (relative to long-term dialysis) [7]. Given the limited supply of donor organs available for transplantation and the growth in the number of ESRD patients requiring kidney transplantation, ensuring the fairness of organ allocation is of paramount importance. It is therefore important to objectively identify barriers, biases and disparities among population subgroups with regard to access to transplantation. Some studies suggest that economic deprivation, female gender, higher age and the presence of comorbidities are inversely associated with access to transplantation [8–10]. However, the association between body mass index (BMI) and access to medical care among ESRD patients has not been extensively examined. There is some evidence [11, 12] to show that the survival benefit from transplantation (relative to dialysis treatment) is similar in obese and non-obese patients; hence, obesity *per se* should not be an exclusion criterion for kidney transplantation. However, few studies have probed the association between BMI and assess to transplantation [13, 14], and none have studied the change over time in BMI after the start of dialysis, with transplantation.

The primary objective of the current study was to determine the association between BMI at the start of dialysis and changes in BMI thereafter with access to transplantation among French adults with ESRD in a large, prospective, nationwide registry. The secondary objective was to assess the relationship between BMI and overall mortality.

## Materials and methods

### Population

The French Renal Epidemiology and Information Network (REIN) registry was created in 2002 and serves as a tool for public health decision support, evaluation and research related to renal replacement therapies for ESRD [15]. The registry relies on a regionally and nationally coordinated network of nephrologists, epidemiologists, patients and public health representatives. Incident patients are reported on the day on which dialysis is initiated. Patients with a diagnosis of acute kidney failure (i.e. patients who recover all or some renal function within 45 days of entry and patients who die within 45 days of entry) are excluded from the registry. By 2009, the REIN registry had grown to include the whole country. Further details are described elsewhere [16]. In the present study, we included 28 110 incident patients (aged 18 to 70) having started dialysis between 2002 and 2011.

### Ethics statement

This prospective study, approved by the French Biomedecine Agency, included patients’ information which have been de-identified directly in the database and before the extraction for analysis. Subjects involved were extracted from the French REIN registry, which received agreement from the Commission Nationale de l’Informatique et des Libertés (CNIL) in 2010. The REIN is registered with the CNIL with 903188 Version 3 number. The study was conducted according to the Declaration of Helsinki guidelines. The clinical and research activities being reported are consistent with the Principles of the Declaration of Istanbul as outlined in the 'Declaration of Istanbul on Organ Trafficking and Transplant Tourism'. None of the transplant donors were from a vulnerable population and all donors or next of kin provided written informed consent that was freely given.

### Data collection

Baseline information included age, gender, primary renal disease, serum albumin, hemoglobin, and the use of erythropoiesis-stimulating agents. The following comorbidities were taken into account: types I and II diabetes, congestive heart failure (New York Heart Association stages I to IV), peripheral vascular disease (Leriche classification stages I–IV), coronary heart disease, stroke or transient ischemic attack, chronic respiratory disease, cardiac dysrhythmia, malignancy, and liver disease (cirrhosis or viral hepatitis). Data on smoking status, mobility status, and severe disabilities that may affect personal autonomy (such as severely impaired vision, amputation, hemiplegia, paraplegia and severe behavioral disorders) were also collected. The context for dialysis initiation was classified as either scheduled hemodialysis, scheduled peritoneal dialysis, and unscheduled dialysis (i.e. emergency initiation of dialysis within 24h after the diagnosis of a life-threatening condition).

Height was recorded at study inclusion. Weight was measured at inclusion and at each dialysis visit, systematically at the end of the dialysis session (dry weight) and recorded in each patient’s medical file. The BMI was calculated as the weight (in kg) divided by the square of the height (in m).

For descriptive analyses, we used both the baseline eGFR (estimated with the CKD EPI equation) and the denormalized GFR (to take account of body surface areas that differed from 1.73 m^2^) estimated with the simplified MDRD equation. For multivariate analyses, the CKD EPI equation was used.

Deaths (during dialysis or after transplantation) and transplantations were registered from the first day of dialysis to the study end date. These analyses reflect all data collected through December 31^st^, 2012.

### Statistical methods

The international World Health Organization classification of adult underweight, overweight and obesity and the European Best Practice Guidelines on Hemodialysis [17] prompted us to analyze the patients’ baseline characteristics according to the following initial BMI strata: <18.5, 18.5–22.9, 23–24.9, 25–29.9, 30–39.9 and ≥40 kg/m^2^.

Continuous and categorical variables are reported respectively as the mean ± standard deviation (SD) or median (25^th^–75^th^ percentiles) and the frequency (percentage). Cubic spline regression analyses provided a graphic evaluation of the relationship between the BMI at the start of dialysis and either overall mortality or access to transplantation. Before drawing the splines, the joint model computation provided tables allowing to derive BMI cutoffs by which the hazard of receiving a renal transplant increased or decreased significantly. Joint models appeared to be more appropriate than time-dependent Cox models for evaluating the association between the change over time in BMI and either transplantation or death. Indeed, the classical time dependent Cox model is only theoretically valid for exogenous time-varying covariates, and it is not optimal when it comes to studying repeated measurements of biomarkers or of other patient parameters [18, 19]. By undertaking a joint model that evaluates both the longitudinal and the survival data simultaneously, we reduce biases and improve precision over simpler approaches [20].

The longitudinal model was modelled by including random spline function, in order to capture the nonlinear subject-specific evolutions. For the event process, we fitted cause-specific Cox regressions. We also additionally included in the linear predictor of the survival submodel the main effect of BMI and its interaction with the failure type indicator (transplanted, death or alive).

Times to outcomes (death, transplantation) were calculated from the date of first dialysis. Non-transplanted living patients were censored at the end of the follow-up: December 31, 2012.

To perform the analysis on the access to transplantation, death was considered a competing event.

For the analysis of the overall ESRD mortality (on dialysis and transplantation), transplantation was not handled as competing event.

For each outcome, six joint models were fitted with different adjustments for explanatory variables. Outcomes were also assessed in three different strata for the baseline BMI (10.5–22.9; 23–29.9; ≥30 kg/m^2^) because we hypothesized that the patients’ “healthcare trajectories” would depend on their BMI at the time of registry entry. Furthermore, we decided to force the variable selection in order to keep age, sex and comorbidities variables in the models given their importance in terms of interpretation. Model 1 was adjusted for age and gender; Model 2 was adjusted for age, gender and comorbidities; Model 3 was adjusted for age, gender and eGFR according to the MDRD equation. Model 4 was adjusted for age, gender and initial treatment (scheduled hemodialysis, unscheduled hemodialysis and peritoneal dialysis); model 5 was adjusted for age, gender and smoking status; model 6 was adjusted for age, gender, comorbidities and albuminemia levels. In these models, BMI was treated as a continuous variable. Adjustments were not performed on laboratory data because of the high number of missing values (36% for serum albumin and 15% for blood hemoglobin).

Sensitivity analyses were performed 1) restricting the sample to patients transplant eligible (without: peripheral vascular disease or stage I-II, coronary heart disease or stage I-II, active malignancy); 2) including transplantation as an adjustment covariate when assessing the association between BMI and mortality.

Hazard ratios (HRs) and their 95% confidence interval (95%CI) are presented below. All statistical tests were two-tailed, and the threshold for statistical significance was set to p<0.05. Analyses were performed with SAS Enterprise Guide 5.1 (SAS Institute, Cary, NC, USA) and R (JMpackage [21]; www.r-project.org) software.

## Results

**Fig 1:**
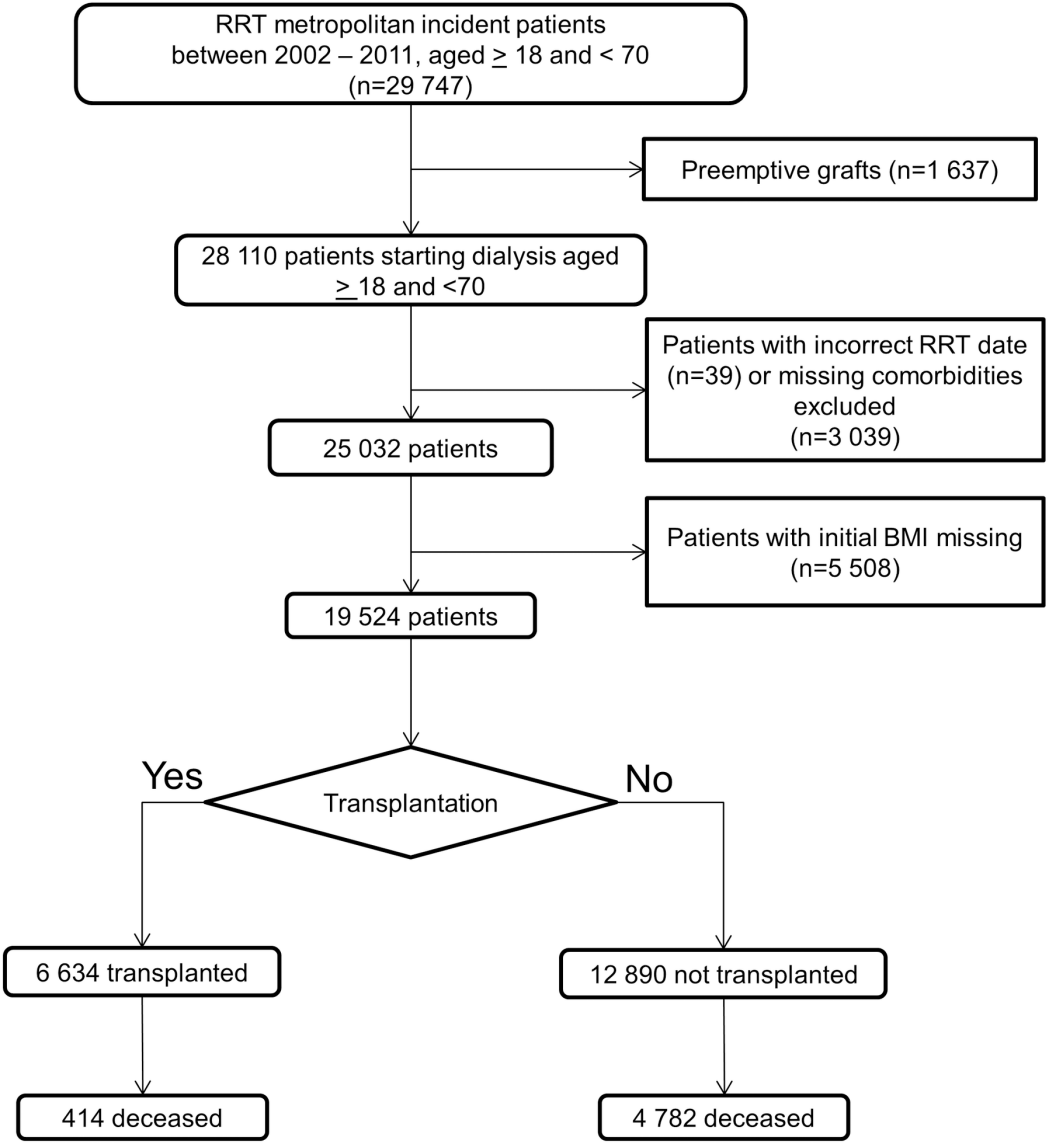
Flow chart for sample selection (RRT: Renal replacement therapy). Relative to the excluded patients, the included patients did not differ significantly in terms of gender and the prevalence of congestive heart failure but were older (+1 year, p<0.001), had a slight lower eGFR at initiation (-0.2ml/min, p = 0.03), were less likely to have diabetes (-2%, p<0.002) and had different patterns of initially treated conditions or primary renal disease (Table A in S1 File).

Between 2002 and 2011, 28110 patients aged 18 to 70 started dialysis. Thirty-nine patients with an incorrect start date, 3 039 patients with missing data on comorbidities and 5 508 with missing data on the baseline BMI were excluded, giving a final sample size of 19 524 (Fig 1.).

### Characteristics of the study population as a function of the BMI category at the start of dialysis

The mean ± SD age and BMI were respectively 54.9 ± 11.9 years and 26.2 ± 6.1 kg/m^2^. Males accounted for 63% of the population; 6% had started dialysis with a BMI <18.5 kg/m^2^ and 23% has started with a BMI ≥30 kg/m^2^ (Table 1).

**Table 1:** Patient characteristics according to the BMI level at start of dialysis.

		Body Mass Index (kg/m^2^)					
	Total (n = 19 524)	<18.5 (n = 1 130)	[18.5–23[ (n = 5 326)	[23–25[ (n = 3 120)	[25–30[ (n = 5 483)	[30–39[ (n = 3 855)	≥40 (n = 610)
** **	** **						
Men	63.3	46.4	62.8	71.5	69.3	57.9	35.9
Age (year)	54.9±11.9	49.7±14.5	51.6±13.5	55.2±11.5	56.9±10.5	57.9±9.6	57.0±9.8
BMI (kg/m^2^)	26.2±6.1	17.1 ± 1.2	21.1 ± 1.2	24.0 ± 0.6	27.3 ± 1.4	33.5 ± 2.6	44.6 ± 4.3
Denormalized eGFR (ml/min)	9.4±7.1	8.3±8.3	8.4±6.8	9.1±6.6	9.5±6.9	10.8±7.1	12.2±7.9
eGFR (CKD EPI) (ml/min)	8.0±6.0	9.2±7.2	8.1±6.4	8.0±6.1	7.8±5.6	8.1±5.5	8.2±5.4
**Primary renal disease**							
Polycystic kidneys	10.2	5.5	11.7	13.1	11.4	6.8	2.5
Glomerulonephritis	17.1	18.3	20.2	18.0	17.2	12.8	9.2
Vascular or hypertensive nephropathy	15.5	14.6	13.2	16.2	17.3	16.3	12.1
Diabetic nephropathy	23.5	10.6	14.3	18.2	24.0	38.9	52.6
Other or unknown	33.7	51.0	40.6	34.6	30.1	25.2	23.6
**Comorbidities and disabilities**	** **						
Diabetes	35.6	16.7	21.2	27.5	38.0	58.0	73.9
Type 2 diabetes	30.3	10.6	14.2	22.2	33.7	53.9	71.0
Congestive heart failure	18.0	18.5	14.3	16.0	17.9	22.8	28.5
Coronary heart disease	17.9	10.8	13.0	16.6	20.2	24.0	21.0
Dysrythmia	10.1	7.1	7.0	8.8	11.4	13.7	13.9
Stroke or transient ischemic attack	7.9	7.4	7.0	7.9	8.0	9.3	7.4
Peripheral vascular disease	15.8	14.0	12.3	14.7	16.8	20.3	17.9
Active malignancy	7.8	11.8	9.0	8.4	7.2	5.7	4.8
Chronic respiratory disease	9.3	12.4	6.4	7.3	7.5	13.9	26.2
Liver disease	5.7	7.5	6.6	6.3	5.6	4.1	3.8
Amputation	2.2	2.2	1.5	1.8	2.2	3.3	4.6
Hemiplegia or paraplegia	1.9	2.4	2.0	1.8	1.8	1.6	1.7
Severely impaired vision	3.1	1.6	2.8	2.8	2.8	4.5	4.8
Severe behavioral disorder	3.3	6.6	3.5	3.0	2.5	3.3	2.7
Mobility	** **						
Totally dependent for transfers	3.1	4.7	2.7	2.5	2.7	3.5	6.6
Need assistance mobility	6.5	9.4	5.8	5.9	5.4	7.2	14.8
Walk without help	90.5	85.9	91.5	91.6	92.0	89.3	78.6
**Predialysis anemia care**							
Hemoglobin <11 g/dl	35.0	30.7	33.6	36.3	37.4	34.7	28.8
Predialysis ESA treatment	49.5	47.2	47.5	48.5	50.8	51.4	52.2
**Nutritional status**	** **						
Albuminemia (g/l)	33.8±6.9	31.9±7.9	33.5±7.1	33.9±7.0	34.4±6.8	34.2±6.1	32.9±6.1
**Initial treatment condition**	** **						
Planned HD	59.1	51.7	56.0	58.8	60.2	63.5	64.0
Unplanned HD	28.4	36.5	30.5	27.7	26.1	26.5	32.1
Peritoneal dialysis	12.5	11.8	13.5	13.5	13.7	10.0	3.9
Weekly number of HD sessions							
1–2	5.9	5.6	5.9	6.4	5.9	5.6	4.4
3	93.0	93.0	93.2	92.7	93.0	93.2	93.4
>3	1.1	1.4	0.9	0.9	1.1	1.2	2.3
Weekly HD session duration > 12 h	7.5	2.9	4.3	6.0	8.1	11.6	17.8

Abbreviations: eGFR, estimated glomerular filtration rate; ESA, erythropoietin stimulating agent; HD, hemodialysis; CKD–EPI, Chronic Kidney Disease Epidemiology Collaboration.

Mean±s.d. or %.

The most prevalent primary renal diseases were diabetic nephropathy (23.5%) and glomerulonephritis (17.1%). Type II diabetes (30.3%), congestive heart failure (18.0%), coronary heart diseases (17.9%) and peripheral vascular diseases (15.8%) were the most prevalent ESRD-associated comorbidities.

Relative to patients with a baseline BMI of between 18.5 and 25 kg/m^2^, patients with the lowest and highest BMI values (<18.5 and ≥40 kg/m^2^) had lower serum albumin and hemoglobin levels, were more likely to be female and were more likely to have chronic respiratory disease. Patients with the highest BMIs were more likely to (i) be older, (ii) have diabetes, congestive heart failure and cancer disease, (iii) be on hemodialysis (vs. peritoneal dialysis) and (iv) be receiving more than three dialysis sessions per week. Lastly, the proportion of patients receiving more than 12 hours of hemodialysis per week increased with the BMI.

### BMI at the start of dialysis, the change over time in BMI and access to kidney transplantation

Thirty-four percent (Table B in S1 File) of the cohort underwent kidney transplantation after a median (25^th^–75^th^ percentiles) of 20.3 (12.0–32.9) months after the initiation of the dialysis. The transplant came from a living donor in 8.2% of the cases.

**Fig 2:**
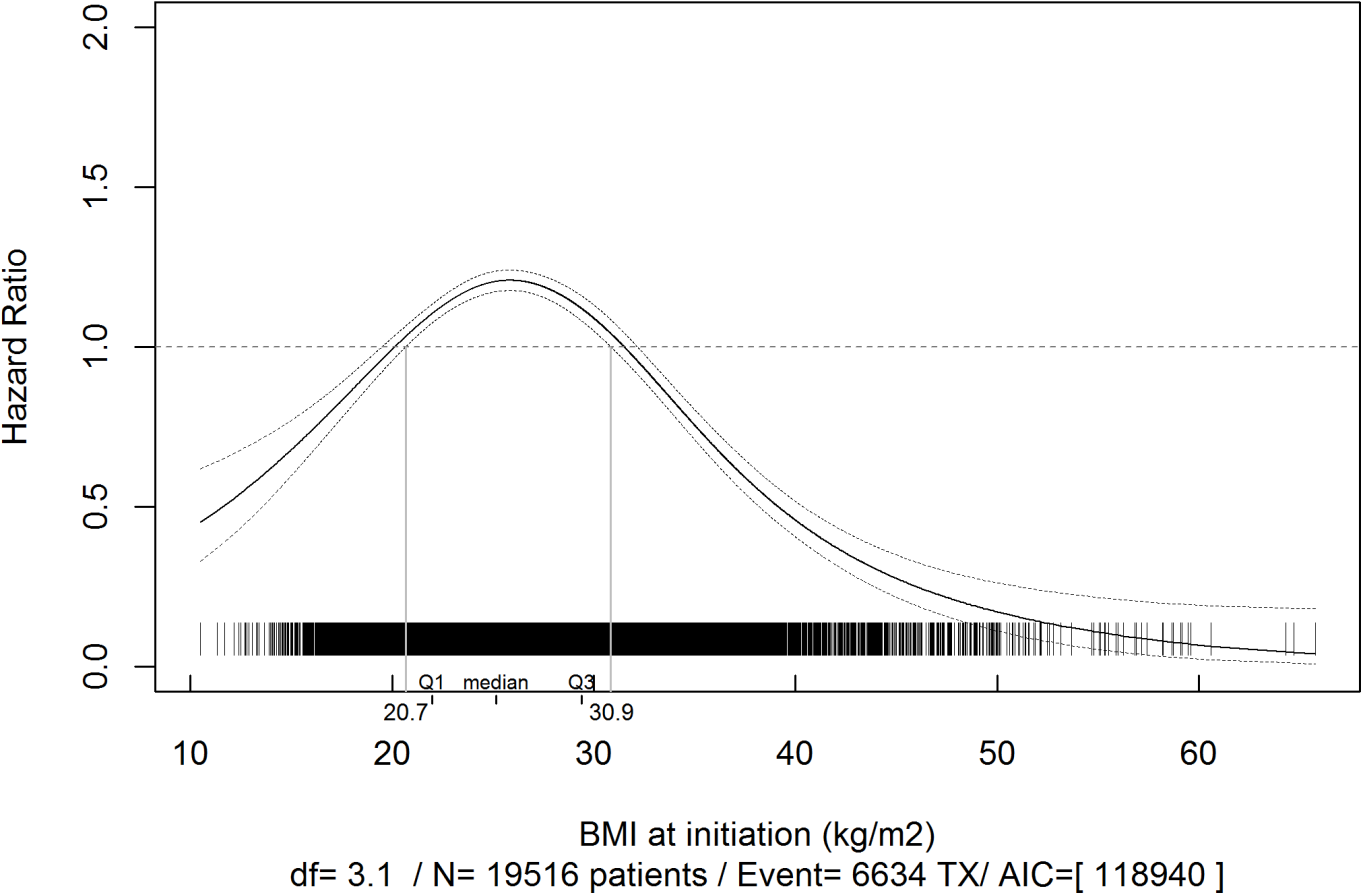
Multivariate associations between body mass index (BMI) at the start of dialysis and access to kidney transplantation. *Adjusted for gender, age, congestive heart failure, diabetes, chronic respiratory disease, coronary heart disease, cardiac dysrhythmia, peripheral vascular disease, active cancer disease and stroke.

According to a multivariate analysis, patients with a BMI ≥31 kg/m^2^ at the start of dialysis are less likely to receive a kidney transplant and this probability decreased as the BMI increased (Fig 2). However, among patients who were obese at the entry in the registry, a 1 kg/m^2^ decrease in BMI during follow-up was associated with a 9 to 11% increase in the likelihood of receiving a kidney transplant (Table 2). The patients with the greatest probability of receiving a kidney transplant were those with an initial BMI of between 21 and 31 kg/m^2^. Moreover, a 1kg/m^2^ decrease in BMI during follow-up among these patients was associated with an additional 2 to 5% increase in the likelihood of transplantation (Table 2).

**Table 2:** Multivariate associations (hazard ratios and 95% confidence intervals derived from joint models) for access to transplantation for BMI in different multivariate joint models stratified on three levels of initial BMI with competing risk of death.

	Hazard Ratio [95% Confidence Interval] Probability of renal transplantation for each 1 kg/m^2^ decrease in the BMI per year		
	10.5–23 (n = 6 456)	23–30 (n = 8 605)	≥30 (n = 4 463)
**Model 1: adjusted for age and gender**	1.01 [0.99–1.02]	1.05 [1.03–1.06]	1.11 [1.09–1.12]
**Model 2: adjusted for age, gender and comorbidities**	1.00 [0.98–1.01]	1.03 [1.02–1.05]	1.10 [1.08–1.11]
**Model 3: adjusted on age, gender and eGFR (CKD–EPI)**	1.02 [1.00–1.04]	1.05 [1.04–1.08]	1.11 [1.10–1.14]
**Model 4: adjusted on age, gender and initial treatment**	1.00 [0.98–1.02]	1.05 [1.03–1.06]	1.11 [1.09–1.12]
**Model 5: adjusted on age, gender and smoking status**	0.99 [0.97–1.01]	1.04 [1.03–1.06]	1.10 [1.09–1.12]
**Model 6: adjusted for age, gender, comorbidities and albuminemia level**	1.01 [0.99–1.04]	1.02 [1.00–1.04]	1.09 [1.06–1.10]

Abbreviations: eGFR, estimated glomerular filtration rate; CKD–EPI, Chronic Kidney Disease Epidemiology Collaboration.

NB: A crude model not possible to compute due to a limitation in the current implementation of the code to do the competing risks.

Hazard ratios were modelled using separate, restricted cubic splines in a Cox regression model.

### BMI at the start of dialysis, the change over time in BMI and overall ESRD mortality

**Fig 3:**
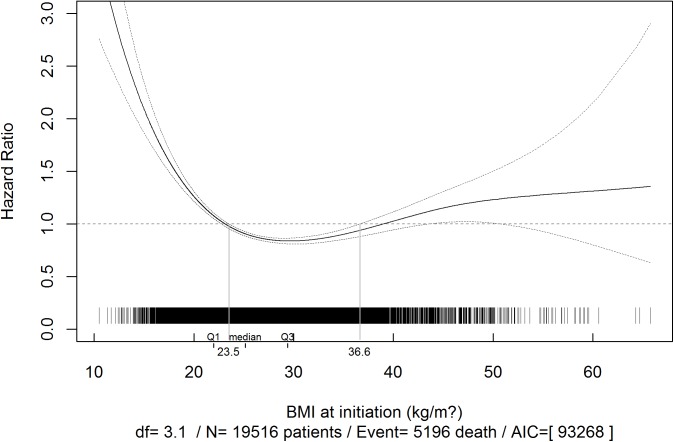
Multivariate associations between body mass index (BMI) at the start of dialysis and overall ESRD mortality. *Adjusted for gender, age, congestive heart failure, diabetes, chronic respiratory disease, coronary heart disease, cardiac dysrhythmia, peripheral vascular disease, active cancer disease and stroke.

Table C in S1 File shows the distribution of deaths by BMI category and by the cause of death. There was an L-shaped relationship between the BMI at the start of dialysis and overall mortality in dialysis or transplantation (Fig 3). Patients with a BMI >37 kg/m^2^ at the start of dialysis had a marginally higher risk of death. This risk barely changed as the BMI increased during the follow-up period (Table 3). Patients with a BMI of between 23 and 37 kg/m^2^ at the start of dialysis had a lower risk of death. For patients with a BMI below 23 kg/m^2^, the risk of death increased steeply as the BMI decreased.

**Table 3:** Multivariate associations (hazard ratios and 95% confidence intervals derived from joint models) for overall mortality for BMI in different multivariate joint models stratified on three levels of initial BMI.

	Hazard Ratio [95% Confidence Interval] Probability of death for each 1 kg/m^2^ decrease in the BMI per year		
	10.5–23 (n = 6 456)	23–30 (n = 8 605)	≥30 (n = 4 463)
**Model 1: adjusted for age and gender**	1.20 [1.18–1.23]	1.06 [1.04–1.09]	0.99 [0.98–1.00]
**Model 2: adjusted for age, gender and comorbidities**	1.19 [1.16–1.22]	1.09 [1.06–1.11]	1.00 [0.98–1.01]
**Model 3: adjusted on age, gender and eGFR (CKD–EPI)**	1.19 [1.16–1.22]	1.06 [1.04–1.09]	0.99 [0.98–1.00]
**Model 4: adjusted on age, gender and initial treatment**	1.20 [1.18–1.23]	1.06 [1.04–1.09]	0.99 [0.98–1.00]
**Model 5: adjusted on age, gender and smocking status**	1.20 [1.18–1.23]	1.06 [1.04–1.09]	0.99 [0.98–1.00]
**Model 6: adjusted on age, gender, comorbidities and albuminemia**	1.18 [1.15–1.22]	1.08 [1.04–1.10]	1.01 [1.00–1.03]

Abbreviations: eGFR, estimated glomerular filtration rate, CKD–EPI, Chronic Kidney Disease Epidemiology Collaboration.

The association between change in BMI over time and the risk of mortality was modulated by the initial BMI (Table 3). For patients with a BMI <30 kg/m^2^ at the start of dialysis, a 1 kg/m^2^ decrease in BMI during follow-up was associated with a higher risk of death. This risk increase was more important for patients with initial BMI between 10.5–23 kg/m^2^ (18–20% higher risk) than for those with a BMI between 23–30 kg/m^2^ (6 to 9% higher risk). Patients with initial BMI above 30 kg/m^2^ experienced a marginally significant 1% decrease in risk of death for each 1 kg/m^2^ weight loss (Table 3).

### Sensitivity analyses

The results did not differ consequently when the study population was restricted to participants who would probably be transplant eligible. Moreover, taking into account transplantation when studying the association between BMI and mortality did not alter the results.

## Discussion

In a French, nationwide, prospective registry, ESRD patients who started dialysis with a BMI >32 kg/m^2^ had a significantly lower likelihood of receiving a kidney transplant. However, this likelihood increased for overweight patients losing weight during follow-up. Our study is the first to show that in these obese patients, a 1kg/m^2^ decrease in BMI after the start of dialysis was associated with a subsequent 9 to 11% improvement in the likelihood of access to kidney transplantation. This limited access to transplantation in obese dialysis patients was in sharp contrast to a relatively good prognosis on dialysis, since the mortality rate was similar to that observed in non-obese patients.

In a *post-hoc* analysis of a prospective cohort of 132 353 adult patients registered for kidney transplantation between 1995 and 2006, Segev et al. [14] analyzed the association between BMI and waiting time for kidney transplantation. They found that the likelihood of receiving a transplant decreased as the degree of obesity increased. In a retrospective analysis of the same population, Gill et al. [13] used multivariate time-to-event analyses to characterize the association between BMI and the likelihood of kidney transplantation. Among men, the BMI threshold associated with a lower likelihood of transplantation was ≥40.0 kg/m^2^ for a transplant from any donor source or from a living donor and ≥35.0 kg/m^2^ for transplants from a deceased donor.

Unlike the two above-mentioned publications, our study also used innovative statistical methods to determine the likelihood of receiving kidney transplantation for each point increase in the BMI point. Gill et al. and Segev et al. used pre-determined BMI cut-offs and assumed that the risk was constant within a given BMI category [13, 14]. Two other studies gathered the data required to assess the association between BMI at the start of dialysis and the likelihood of kidney transplantation in either a broad ESRD population [22] or diabetic ESRD patients [23]. However, these researchers stratified their analyses on BMI categories and thus were unable to determine the effect of BMI on access to kidney transplantation.

There were significant differences in comorbid conditions among patient included compared to those excluded from the statistical analyses. To the small absolute differences, the significativity was probably related to the statistical power.

Kidney transplantation is the treatment of choice for ESRD patients. The ideal public health outcome is to make this treatment available to all eligible patients, irrespective of age, gender, race or even socioeconomic status. There are still marked disparities in access to kidney transplantation; with a view to developing potentially corrective interventions, it is of the utmost importance to identify factors and pathways that influence access to transplantation. Previous studies have identified a number of disparities that cannot be fully explained by medical criteria (including ethnicity [24], gender [8, 25], age [24], primary renal disease [9, 24] and comorbidities [23]). The effect of obesity on access to kidney transplantation has not been extensively characterized, and the present study is the first to assess the effect of the changes of BMI over time on the access to kidney transplantation.

There is a number of possible explanations for poorer access to kidney transplantation in obese patients. Until recently, obesity was associated with an increased risk of delayed graft function [26], wound complications [27], longer hospital admissions for deceased donor transplantation [28], hospital readmission [29], and new-onset diabetes after transplantation [30]. Obesity was therefore associated with lower survival, higher transplantation costs and decreased quality of life. In a recent meta-analysis of 21 studies and a total of 9296 patients with ESRD [31], Nicoletto et al. observed that before the year 2000, obesity was a risk factor for graft loss, cardiovascular death and all-cause mortality. However, obese patients transplanted after 2000 had much the same graft loss and survival rates as non-obese patients. In agreement with these literature results, we found that a BMI above 38 kg/m^2^ at the start of dialysis was not associated with higher risk of death. Among obese patients, there was only a weak association between an increase in BMI after the initiation of dialysis and the risk of death.

Some (but not all) of today’s practice guidelines suggest that obesity status should influence a patient’s selection for kidney transplantation. The American Society of Transplantation recommends a supervised weight-loss regimen, including a low-calorie diet, behavioral therapy, and a physical activity plan to achieve a target BMI <30 kg/m^2^ prior to kidney transplantation [32]. The Canadian Transplant Society has released similar guidelines, although a statement on the lack of data for excluding obese patients appears before a recommendation for weight reduction [33]. In contrast, the European Best Practice Guidelines did not include specific recommendations on obesity and kidney transplantation [34]. In view of the lack of clear evidence, acceptable BMI limits for kidney transplant candidates vary greatly from one transplant center to another. Moreover, our results call these recommendations into question, since a decreasing BMI (whether intentional or not) was associated with increasing mortality in dialysis patients. Unfortunately, the REIN database does not mention whether a patient underwent or not bariatric surgery. As this intervention is more and more an alternative for weight loss in severely obese patients, further studies should focus on the effect of weight loss following bariatric surgery on mortality or access to kidney transplantation.

Our study had several strengths. Firstly, by examining the correlates of a change in weight over time, we allowed a better control of unmeasured confounders and the change over time in BMI. Secondly, the follow-up period was relatively long. Thirdly, our analyses were adjusted for the presence of chronic disease and smoking status, which are associated with both decreased BMI and an increased risk of death. Reverse causation (owing to inadequate control for these two confounding factors) might distort the true relationship between BMI and mortality risk.

Nevertheless, our study also had several limitations. We used BMI as a proxy for obesity, although BMI only indirectly reflects the metabolic effects of an increased fat mass. Moreover, BMI does not fully reflect some age-related changes, such as a change in body fat mass or a decrease in muscle mass with age. In other studies of non-dialyzed patients [35], all-cause mortality and cardiovascular mortality were more closely related to a high hip-to-waist ratio than to a high BMI *per se*. Failure to control for unintentional weight loss could result in residual confounding. Lastly, given the participant characteristics mentioned above, it is not yet known whether our findings can be generalized to other populations of dialysis patients.

This study has implications for clinical practice, research and health policy. Obese individuals are often subject to various forms of social stigma and discrimination. Transplant-related decisions should be guided by medical considerations and the patients’ best interests, regardless of their BMI. Appropriate weight-loss or weight maintenance counseling should be a routine part of the transplant workup. However, interventional studies should test the effectiveness and safety of weight-loss programs in obese patients seeking kidney transplantation for ESRD.

## Supporting information

S1 File Supplemental material, material and methods. **Table A:** Comparison of characteristics of included and excluded patients. **Table B:** Renal transplantation according to the BMI level at the start of dialysis. **Table C:** Causes of death according to the BMI level at the start of dialysis.
(DOC)
