# Supplementary material for: Obesity and access to kidney transplantation in patients starting dialysis: A prospective cohort study
Source: PLoS One. 2017 May 11;12(5):e0176616. doi: 10.1371/journal.pone.0176616 (PMC5426620; doi:10.1371/journal.pone.0176616)
Supplement: S1 File — Table A: Comparison of characteristics of included and excluded patients. Table B: Renal transplantation according to the BMI level at the start of dialysis. Table C: Causes of death according to the BMI level at the start of dialysis. (DOC) [file pone.0176616.s001.doc]

**Supporting information, Methods**

In follow-up studies, the primary aim is to analyze the time until a prespecified event of interest occurs and the association with multiple independent prognostic factors. Among those factors, one may consider **time-dependent covariates**, i.e for which the value is not constant during follow-up. External or exogenous covariates are associated with the rate of event over time but are not affected by the occurrence of event; for example, environmental factors such as air pollution. On the other hand, internal or endogenous covariates, like biomarkers and clinical parameters, arise as time-dependent measurements taken on the subjects under study. They typically require the survival of the subject for their existence. Thus, when the event of interest is death of the subject, their path carries direct information about the event time. They are also measured with “error” due to biological variation induced by the patient. Finally, their complete pathway is not fully observed but measured intermittently for specific occasions like dialysis schedules. The Cox model can be extended to handle exogenous time-dependent covariates. However, despite its flexibility the extended Cox model is not appropriate when the time-dependent covariates are of endogenous nature^1^. In this model, time-dependent covariates are assumed to change value at the follow-up visits and remain constant in the time interval in between these visits. The model postulates that the hazard for an event, at any time point is associated with the extrapolated value of the covariate at the same time point. When primary interest is the association between endogenous time-dependent covariates and survival, an alternative modelling framework has been introduced, known as the **joint-modelling framework for longitudinal and time-to-event data^2,3^**. In these models, a survival submodel is couple with a longitudinal submodel that postulates a suitable mixed-effects model to describe the subject-specific time evolutions and both submodels are linked using a shared latent structure^4^. Parameters of joint models are typically estimated by maximizing the joint likelihood. This approach is preferable to separate analyses, both to make optimal use of the available information and to obtain unbiased estimates of the model parameters. Many extensions of these models have been proposed to take into account various association structure between the longitudinal and event outcomes.

Reference List

(1) Rizopoulos D, Takkenberg JJ. Tools & techniques--statistics: Dealing with time-varying covariates in survival analysis--joint models versus Cox models. *EuroIntervention* 2014;10(2):285-288.

(2) Boucquemont J, Heinze G, Jager KJ, Oberbauer R, Leffondre K. Regression methods for investigating risk factors of chronic kidney disease outcomes: the state of the art. *BMC Nephrol* 2014;15:45.

(3) Asar O, Ritchie J, Kalra PA, Diggle PJ. Joint modelling of repeated measurement and time-to-event data: an introductory tutorial. *Int J Epidemiol* 2015;44(1):334-344.

(4) Rizopoulos D. JM: An R package for the joint modelling of longitudinal and time-to-event data. *Journal of Statistical Software* 2010;35(9):1-33.

**Table A: Comparison of characteristics of included and excluded patients**

|  | Included (n=19 524) | Excluded (n=8 586) | p |
| --- | --- | --- | --- |
| Male, % | 63.3 | 64.0 | 0.2 |
| Age, years | 54.9 ± 11.9 | 53.9 ± 12.5 | 0.0001 |
| eGFR (CKEPI) (ml/min) | 8.0 ± 6.0 | 8.2 ± 6.4 | 0.03 |
| **Primary renal disease, %** |  |  | 0.0001 |
| Polycystic kidneys | 10.2 | 9.4 |  |
| Glomerulonephritis | 17.1 | 15.0 |  |
| Vascular or hypertensive nephropathy | 15.5 | 15.3 |  |
| Diabetic nephropathy | 23.5 | 21.3 |  |
| Other or unknown | 33.7 | 34.6 |  |
| **Comorbidities and disabilities, %** |  |  |  |
| Diabetes | 35.6 | 37.6 | 0.002 |
| Coronary heart disease | 17.9 | 18.2 | 0.6 |
| **Initial treatment condition, %** |  |  | 0.0001 |
| Planned HD | 59.1 | 63.1 |  |
| Unplanned HD | 28.4 | 26.2 |  |
| Peritoneal dialysis | 12.5 | 10.7 |  |

**Table B: Access to transplantation according to the BMI level at the** start of dialysis

|  |  | **Total** |  | **BMI at the start of dialysis** | | | | | | | | | | |
| --- | --- | --- | --- | --- | --- | --- | --- | --- | --- | --- | --- | --- | --- | --- |
|  |  |  |  |  |  |  |  |  |  |  |  |  |  |  |
|  |  |  |  | < 18.5 |  | 18.5-22.9 |  | 23.0-24.9 |  | 25.0-29.9 |  | 30.0-39.9 |  | ≥ 40.0 |
|  |  |  |  |  |  |  |  |  |  |  |  |  |  |  |
| **N** |  | 19 524 |  | 1 130 |  | 5 326 |  | 3 120 |  | 5 483 |  | 3 855 |  | 610 |
|  |  |  |  |  |  |  |  |  |  |  |  |  |  |  |
| **Transplantations, n (%)** |  | 6634 (34.0) |  | 342 (30.3) |  | 2147 (40.3) |  | 1218 (39.0) |  | 1960 (35.8) |  | 917 (23.8) |  | 50 (8.2) |
|  |  |  |  |  |  |  |  |  |  |  |  |  |  |  |
| **Origin of the graft among the 6634 transplanted patients, n (%)** | | | | |  |  |  |  |  |  |  |  |  |  |
|  |  |  |  |  |  |  |  |  |  |  |  |  |  |  |
| Living donor |  | 542 (8.2) |  | 41 (12.0) |  | 191 (8.9) |  | 101 (8.3) |  | 143 (7.3) |  | 61 (6.6) |  | 5 (10.0) |
|  |  |  |  |  |  |  |  |  |  |  |  |  |  |  |
| Deceased donor |  | 6087 (91.8) |  | 299 (87.4) |  | 1955 (91.1) |  | 1117 (91.7) |  | 1815 (92.7) |  | 856 (93.4) |  | 45 (90.0) |

**Table C: Causes of death according to the BMI level at the** start of dialysis

|  |  | **Total** |  | **BMI at the start of dialysis** | | | | | | | | | | |
| --- | --- | --- | --- | --- | --- | --- | --- | --- | --- | --- | --- | --- | --- | --- |
|  |  |  |  |  |  |  |  |  |  |  |  |  |  |  |
|  |  |  |  | < 18.5 |  | 18.5-22.9 |  | 23.0-24.9 |  | 25.0-29.9 |  | 30.0-39.9 |  | ≥ 40.0 |
|  |  |  |  |  |  |  |  |  |  |  |  |  |  |  |
| **N** |  | 19 524 |  | 1 130 |  | 5 326 |  | 3 120 |  | 5 483 |  | 3 855 |  | 610 |
|  |  |  |  |  |  |  |  |  |  |  |  |  |  |  |
| **Deaths, n (%)** |  | 5196 (26.6) |  | 381 (33.7) |  | 1296 (24.3) |  | 750 (24.0) |  | 1445 (26.3) |  | 1108 (28.7) |  | 216 (35.4) |
|  |  |  |  |  |  |  |  |  |  |  |  |  |  |  |
| **Causes of death, n (%)** |  |  |  |  |  |  |  |  |  |  |  |  |  |  |
|  |  |  |  |  |  |  |  |  |  |  |  |  |  |  |
| Disease of the circulatory system |  | 1299 (25.0) |  | 69 (18.2) |  | 293 (22.6) |  | 185 (24.7) |  | 305 (26.6) |  | 302 (27.3) |  | 65 (30.1) |
|  |  |  |  |  |  |  |  |  |  |  |  |  |  |  |
| Cancer |  | 645 (12.5) |  | 47 (12.3) |  | 208 (16.1) |  | 111 (14.8) |  | 143 (12.5) |  | 88 (7.9) |  | 13 (6.0) |
|  |  |  |  |  |  |  |  |  |  |  |  |  |  |  |
| Infection |  | 613 (11.8) |  | 34 (8.9) |  | 131 (10.1) |  | 86 (11.5) |  | 144 (12.6) |  | 151 (13.6) |  | 26 (12.0) |
|  |  |  |  |  |  |  |  |  |  |  |  |  |  |  |
| Other |  | 2234 (43.0) |  | 202 (53.0) |  | 551 (42.5) |  | 303 (40.3) |  | 461 (40.3) |  | 496 (44.8) |  | 102 (47.2) |
|  |  |  |  |  |  |  |  |  |  |  |  |  |  |  |
| Missing |  | 405 (7.7) |  | 29 (7.6) |  | 113 (8.7) |  | 65 (8.7) |  | 92 (8.0) |  | 71 (6.4) |  | 10 (4.6) |

BMI: Body mass index
